# The aspartate aminotransferase-to-platelet ratio and the evaluation of non-alcoholic fatty liver disease

**DOI:** 10.1093/gastro/gou080

**Published:** 2014-11-03

**Authors:** Elliot B. Tapper

**Affiliations:** Division of Gastroenterology, Beth Israel Deaconess Medical Center, Boston, Mass.,USA.

Dear Editor,

We thank Agilli *et al.* and Kayadibi *et al.* for their interest in our manuscript [1]. Taken together, their letters raise points of clarification regarding the utility and general applicability of the ‘aspartate aminotransferase-to-platelet ratio index’ (APRI) test in the evaluation of liver fibrosis.

Our first comment is that their concern over confounders of the APRI unrelated to liver disease is valid. Indeed, confounders would diminish the ability of APRI to predict advanced liver fibrosis, leading to false negatives. Given that our results were significant, this insight strengthens our findings. Also, in defense of APRI, most other non-invasive predictors of liver fibrosis are vulnerable to confounding by extraneous conditions, which could instead lead to false positives. The ‘hepascore’ and ‘fibrotest’, for example, utilizes bilirubin (confounded by Gilbert's) and Gamma-Glutamyl Transferase (GGT) (confounded by cholestasis) [2].

Second, Kayadibi *et al.* posit that non-invasive tests could replace liver biopsy, but the clinical outcomes and cost-effectiveness of this strategy in this population have not been evaluated. For the time being, clinicians should seriously consider biopsy in patients with non-alcoholic fatty liver disease. This is especially true for those with intermediate or contradictory results from non-invasive tests. Furthermore, guidelines recommend liver biopsy to diagnosis non-alcoholic steatohepatitis [3]. Like the authors, we also agree that non-invasive tests to determine the presence of steatohepatitis and fibrosis are advancing.

Third, both groups raise issues regarding test cut-offs. Specifically, they argue that the upper limits of normal AST differ between both laboratories and genders and that APRI’s cut-off value could be 1.5. In general, arbitrary cut-offs reduce the clinical and statistical power of laboratory tests. Additionally, we agree that the prevalence of significant liver disease beneath the so-called ‘upper limits of normal’ (ULN) should not be ignored [4, 5]. APRI is just one helpful piece of information that assists in clinical decision-making.

In principle, one could optimize the characteristics of the APRI test by adjusting its parameters. In response to the specific criticisms, we show below how, the lower the cut-off, the higher the sensitivity and the lower the specificity of APRI (Table 1). However, there are many powerful tests for the non-invasive prediction of fibrosis [6, 7]. APRI's comparative advantage is its ease of use. The power of APRI could be improved, but at the cost of complexity. One could render it into a continuous variable with a beta coefficient or adjust the precise cut-off to some fraction other than our figure of 1.0, or by using different AST cut-offs. Yet, no matter how far optimized, it is unlikely to compete for accuracy with more sophisticated tests [6]. The virtue of APRI is—and always has been—its simplicity [8].

**Table 1.** The effect of AST upper limit of normal cut-off on the performance of the AST-to-platelet ratio

	Adjusted OR for significant fibrosis (95% CI)	Sensitivity % (95% CI)	Specificity % (95% CI)
AST cut-off			
26 IU/L	5.69 (2.82–11.7)	59.1 (43.3–73.3)	81.3 (75.2–86.2)
40 IU/L	3.85 (1.55–9.59)	30.0 (17.2–45.4)	92.8 (88.2–95.8)
49 IU/L	3.75 (1.25–10.8)	15.9 (7.2–30.7)	95.2 (91.1–97.6)
APRI cut-off			
1.0	3.85 (1.55–9.59)	30.0 (17.2–45.4)	92.8 (88.2–95.8)
1.5	1.48 (0.16–3.42)	6.82 (1.51–18.7)	97.1 (93.9–98.9)
2.0	1.28 (0.19–25.2)	2.27 (0.38–12.1)	97.6 (94.5–99.2)

CI = confidence interval; OR = odds ratio
